# DSP Toxin Distribution across Organs in Mice after Acute Oral Administration

**DOI:** 10.3390/md19010023

**Published:** 2021-01-08

**Authors:** M. Carmen Louzao, Paula Abal, Celia Costas, Toshiyuki Suzuki, Ryuichi Watanabe, Natalia Vilariño, Ana M. Botana, Mercedes R. Vieytes, Luis M. Botana

**Affiliations:** 1 Departamento de Farmacologia, Facultad de Veterinaria, Universidad de Santiago de Compostela, Campus Universitario, 27002 Lugo, Spain; paula.abal@usc.es (P.A.); celia.costas@rai.usc.es (C.C.); natalia.vilarino@usc.es (N.V.); luis.botana@usc.es (L.M.B.); 2 Fisheries Technology Institute, National Research and Development Agency, Japan Fisheries Research and Education Agency, Yokohama 236-8648, Japan; tsuzuki@affrc.go.jp (T.S.); rwatanabe@affrc.go.jp (R.W.); 3 Departamento de Quimica Analítica, Facultad de Ciencias, Universidad de Santiago de Compostela, Campus Universitario, 27002 Lugo, Spain; anamaria.botana@usc.es; 4 Departamento de Fisiologia, Facultad de Veterinaria, Universidad de Santiago de Compostela, Campus Universitario, 27002 Lugo, Spain; mmercedes.rodriguez@usc.es

**Keywords:** dinophysistoxin-1, dinophysistoxin-2, LC/MS/MS, okadaic acid, toxicokinetic

## Abstract

Okadaic acid (OA) and its main structural analogs dinophysistoxin-1 (DTX1) and dinophysistoxin-2 (DTX2) are marine lipophilic phycotoxins distributed worldwide that can be accumulated by edible shellfish and can cause diarrheic shellfish poisoning (DSP). In order to study their toxicokinetics, mice were treated with different doses of OA, DTX1, or DTX2 and signs of toxicity were recorded up to 24 h. Toxin distribution in the main organs from the gastrointestinal tract was assessed by liquid chromatography-mass spectrometry (LC/MS/MS) analysis. Our results indicate a dose-dependency in gastrointestinal absorption of these toxins. Twenty-four hours post-administration, the highest concentration of toxin was detected in the stomach and, in descending order, in the large intestine, small intestine, and liver. There was also a different toxicokinetic pathway between OA, DTX1, and DTX2. When the same toxin doses are compared, more OA than DTX1 is detected in the small intestine. OA and DTX1 showed similar concentrations in the stomach, liver, and large intestine tissues, but the amount of DTX2 is much lower in all these organs, providing information on DSP toxicokinetics for human safety assessment.

## 1. Introduction

Okadaic acid (OA) and dinophysistoxins (DTXs) are marine lipophilic phycotoxins globally distributed and produced by benthic and planktonic dinoflagellates of the genera *Prorocentrum* and *Dinophysis* [1,2], with the species of the genus *Dinophysis* being the main source of toxins in the marine trophic chain [3,4]. This group of toxins includes a wide range of molecules, with dinophysistoxin-1 (DTX1) [1] and dinophysistoxin-2 (DTX2) [5] being the main structural analogs of OA [6]. Harmful Algal Blooms (HABs) cause the accumulation of these toxins in edible filter-feeding shellfish, the ingestion of which by human consumers leads to diarrheic shellfish poisoning (DSP) [7]. This gastrointestinal illness is characterized by symptoms such as diarrhea, nausea, and vomiting that begin from 30 min to a few hours after ingestion of the toxic shellfish [8]. OA and DTX2 are routinely found in mussels, clams, and oysters in the Atlantic coast [9,10], while DTX1 is commonly found in Japan [11]. The presence of these toxins is also associated with important economic consequences for producing areas [12].

OA and DTXs are inhibitors of protein phosphatases (PP), mainly PP2A [13], with different potency [6,14,15]. PPs are important modulators of enzyme activity and cell signaling pathways [16]; however, the OA-dependent molecular mechanisms leading to diarrhea are not fully elucidated [15,17,18]. Some studies have indicated that the target organ of OA and DTXs is the small intestine [19,20,21], but the mode of toxicity seems complex and diverse [22]. OA could have targets, other than PPs, involved in the diarrhetic process [23]. Some studies revealed that modulation of neuropeptide levels induced by OA may be the key triggers of diarrhea [24].

Acute Refence Dose (ARfD) and Lowest Observed Adverse Effect Level (LOAEL) have been established in humans, for which the values are 0.3 and 0.8 μg OA equivalents/kg body weight (bw) for adults, respectively [25]. Nevertheless, the toxicological database for the OA group of toxins is limited and comprises mostly studies on their intraperitoneal acute toxicity in mice. A recent study based on the oral lethal doses 50 (LD_50_) in mice showed that DTX1 is the most toxic analog and that DTX2 is the least toxic one [15], suggesting a reevaluation of the Toxicity Equivalency Factor (TEF) values previously established by the European Food Safety Authority (EFSA) considering intraperitoneal LD_50_ [25].

It has been seen that these toxins are easily absorbed orally in a short period of time [15,19,26,27]. However, what happens to OA and its analogs in the body remain to be determined. As human exposure to OA and DTXs is exclusively by ingestion, the aim of this study was to characterize their kinetics following oral administration to mice. Thus, we perform the first comparative toxicokinetic study of OA, DTX1, and DTX2 measuring the toxins in gastrointestinal tissues by LC/MS/MS analysis, the official method for detection of the lipophilic toxins group, where OA-group toxins are also included [28].

## 2. Results

### 2.1. Lethality and Symptoms

In vivo studies were performed following an optimized 4-level up and down procedure where the toxins were administered individually by oral gavage to female mice. The lethality 24 h after oral gavage administration was 67% in mice treated with 1000 µg/kg OA or DTX1. At the same time, point lethality was 0% in mice treated with 1000 µg/kg DTX2. This clearly confirms that DTX2 is less toxic than OA or DTX1. Moreover, DTX1 was more toxic than OA since lethality was 60% in mice treated with 500 µg/kg DTX1 and 40% in mice treated with the same dose of OA (Table 1). These data agree with the previously determined LD_50_ for DTX1 (487 µg/kg), OA (760 µg/kg), and DTX2 (2262 µg/kg) [15,27].

The mice were observed during the whole experiment, and toxicity signs were recorded. Diarrhea and nonspecific symptoms were quickly evident in both OA and DTX1 treated mice (Table 2). However, mice administered with the less toxic compound DTX2 showed individual variability, and nonspecific symptoms such as piloerection, squint-eyes, spasms, and posture on hind legs were less common.

### 2.2. LC/MS/MS Analysis

**Figure 1:**
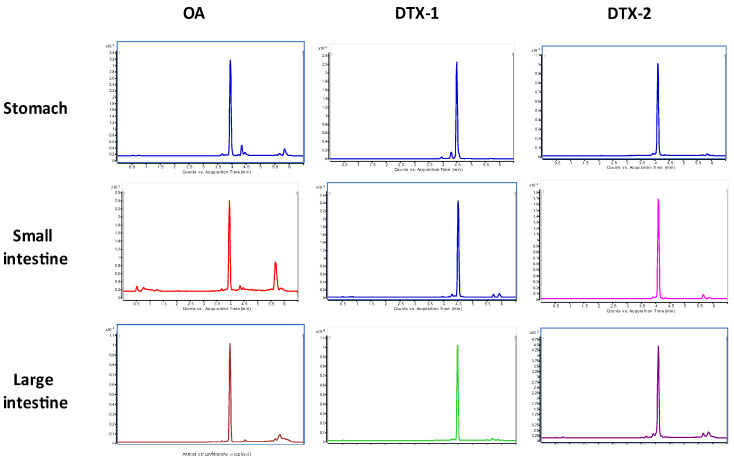
Total ion chromatogram of OA, DTX1, and DTX2 of the stomach, small intestine, and large intestine samples.

In those kinds of studies, after mice death or euthanasia, organs from the gastrointestinal tract were collected to quantify DSP toxins by LC/MS/MS, both in the tissue itself and in the content of some of the organs (contents of the stomach and the small and large intestines). The screening of OA, DTX1, and DTX2 across different organs is presented in Figure 1.

The organs belonging to the digestive tract showed a dose-dependent toxin concentration in most cases. Toxins were more concentrated in stomach tissue and in descending order in the large intestine, small intestine, and liver.

**Figure 2:**
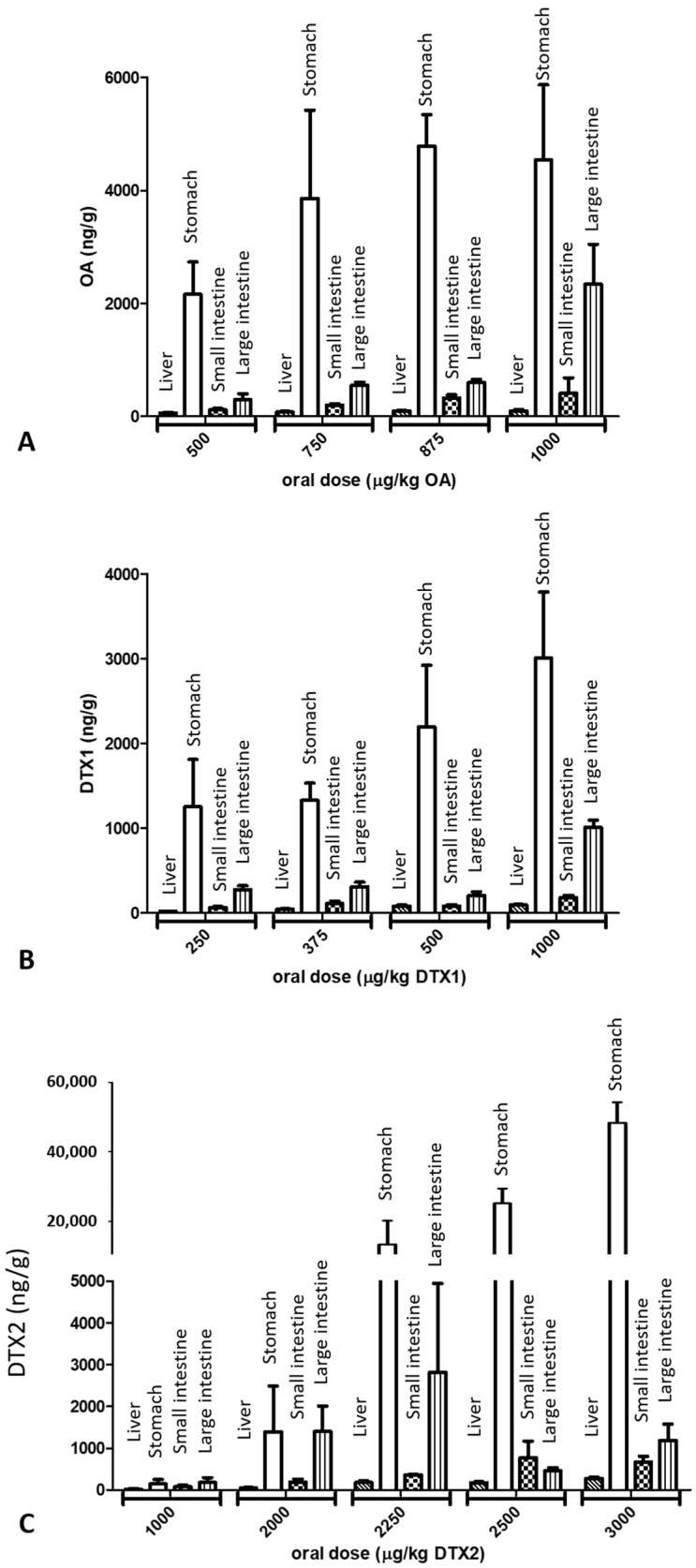
Toxin concentration in different tissues of the digestive organs (ng/g) according to oral dose administered to mice: (**A**) okadaic acid, (**B**) dinophysistoxin-1, and (**C**) dinophysistoxin-2, reported as mean ± SEM.

The highest amount of toxins was found in the stomach (Figure 2). The less toxic compound DTX2 quantified in this organ encompassed much lower values at the doses of 1000 and 2000 µg/kg of DTX2 bw but reached very high concentrations in tissue at the highest doses (2250–3000 µg/kg) (Figure 2C). Statistically significant differences were obtained between OA and DTX2.

The large intestine was another organ with high toxin content, especially OA. The concentration of DTX2 was low, even at the highest doses administered (Figure 2C), and was statistically different from OA. It should be noted that the contents of the three toxins in liver were very low.

When we compared the results of the same dose, we found that mice treated with OA and DTX1 had similar concentrations of toxins in stomach tissue while the amount of DTX2 was much lower (Table 3). Similar results were registered in the liver and large intestine. It is interesting that, in the small intestine, the concentration of the most toxic compound DTX1 was markedly inferior to OA, around 2 times less.

**Figure 3:**
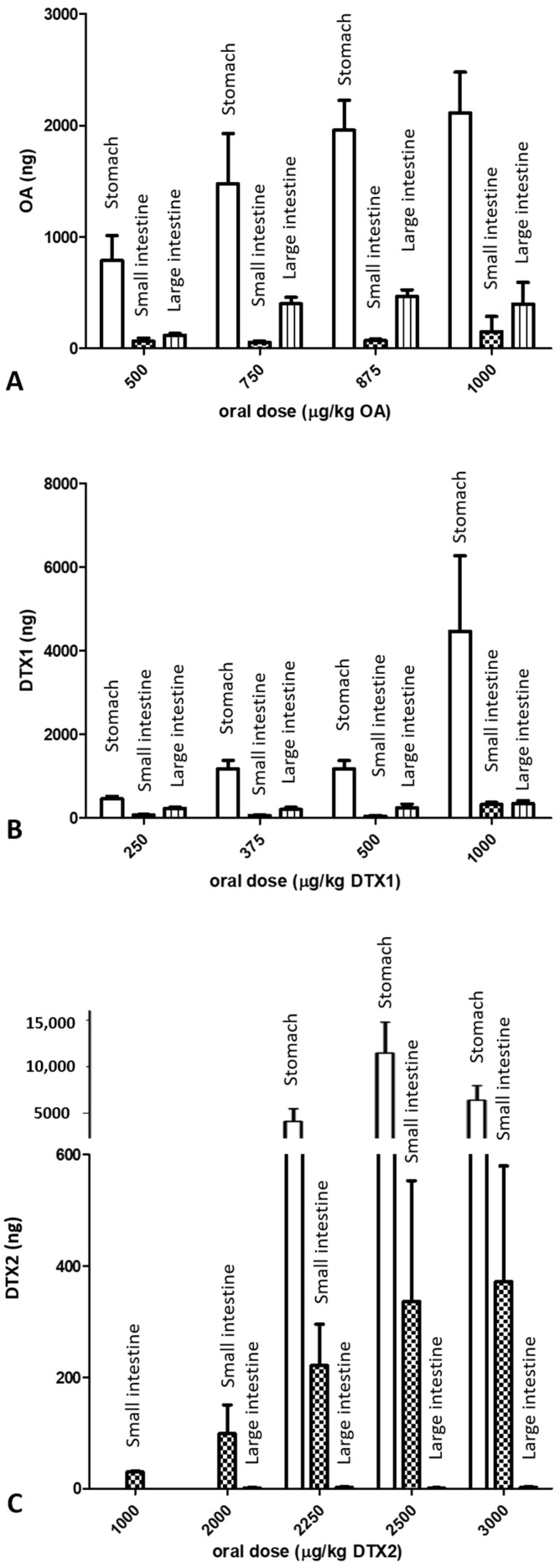
Total toxin (ng) detected in different contents of the digestive organs according to oral dose administered to mice: (**A**) okadaic acid, (**B**) dinophysistoxin-1, and (**C**) dinophysistoxin-2, reported as mean ± SEM.

The contents of the stomach, small intestine, and large intestine were collected from mice treated with each of the three toxins 24 h after administration (Figure 3). The highest amount of OA and DTX1 was found in the stomach content followed by the large intestine and small intestine contents. However, mice treated with DTX2 showed almost a lack of toxin in large intestine content (Figure 3C).

The comparison between toxins (Table 4) indicated that 24 h after administration of 1000 µg/kg of toxin, the amount of DTX1 in the stomach content and small intestine fluids is twofold higher compared to OA. Additionally, DTX1 is tenfold higher compared to DTX2 in the small intestine content, although these differences were not significant. There was a small difference in OA and DTX1 data in the large intestine content. Nevertheless, DTX2 was almost absent in fluids collected form the large intestine. Significant differences between DTX1 and DTX2 were registered.

**Figure 4:**
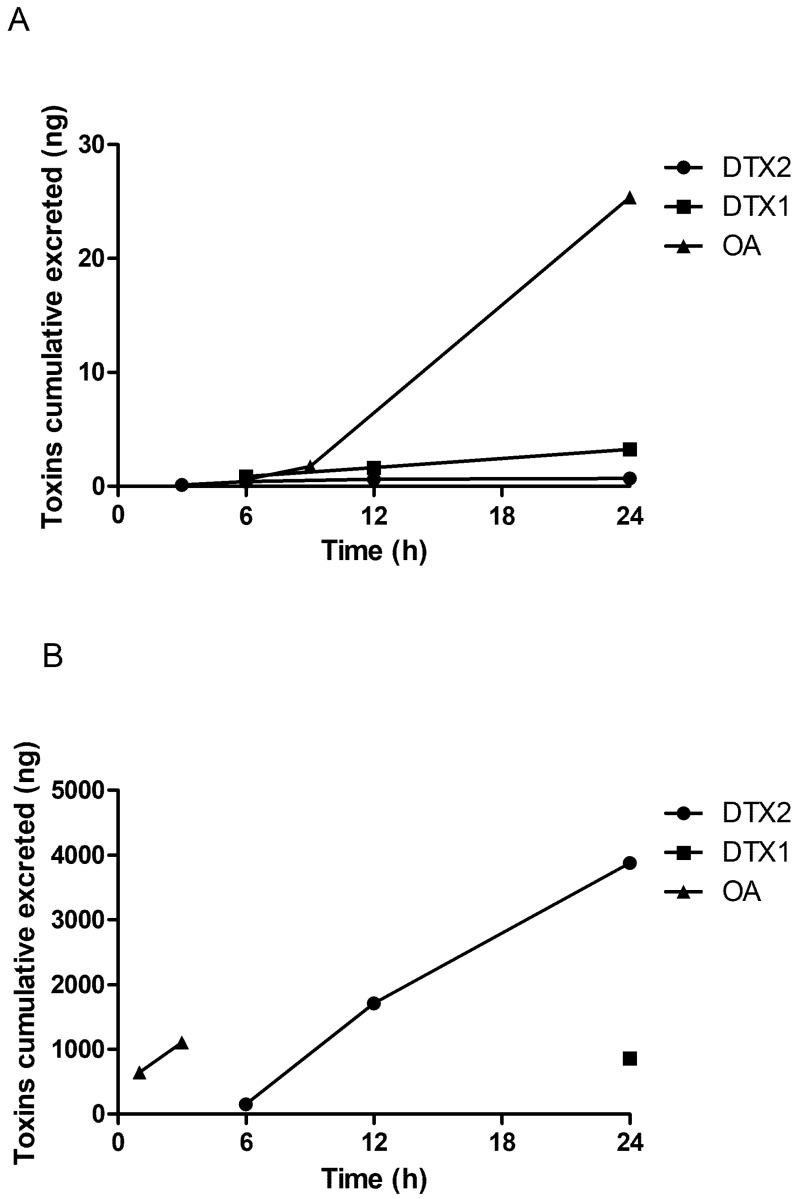
Time course of OA, DTX1, or DTX2 cumulatively excreted (ng) in urine (**A**) and feces (**B**): mice received a dose of 1000 µg/kg of each toxin.

The cumulative toxin excreted in urine and feces after doses of 1000 µg/kg OA, DTX1, or DTX2 in mice are summarized in Figure 3. In urine, OA increased with time up to 24 h (Figure 4A). However, the main excretion of toxins was in feces. OA was excreted with the first diarrheic feces 1 and 3 h after toxin administration. Meanwhile, DTX2 was detected in samples of feces collected 6, 12, and 24 h after toxin administration.

**Figure 5:**
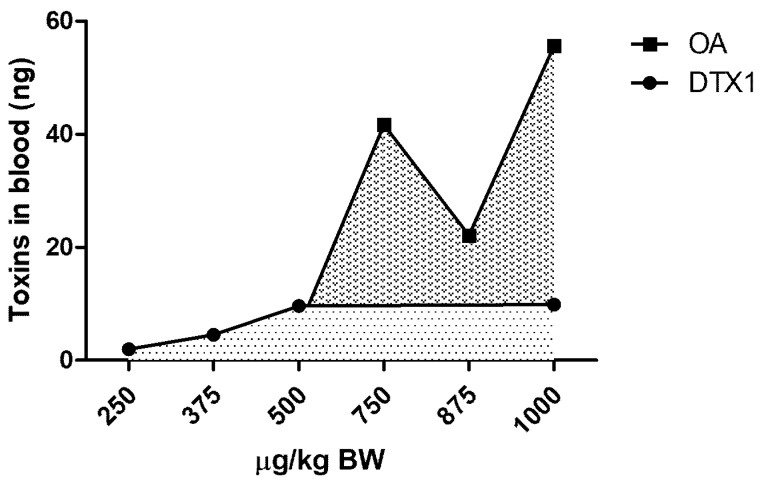
Plasma amount of toxins in mice 24 h after oral gavage administration of each dose of OA or DTX1.

Twenty-four hours following gavage administration of OA and DTX1, measurable concentrations of toxins were found in the blood (Figure 5).

**Figure 6:**
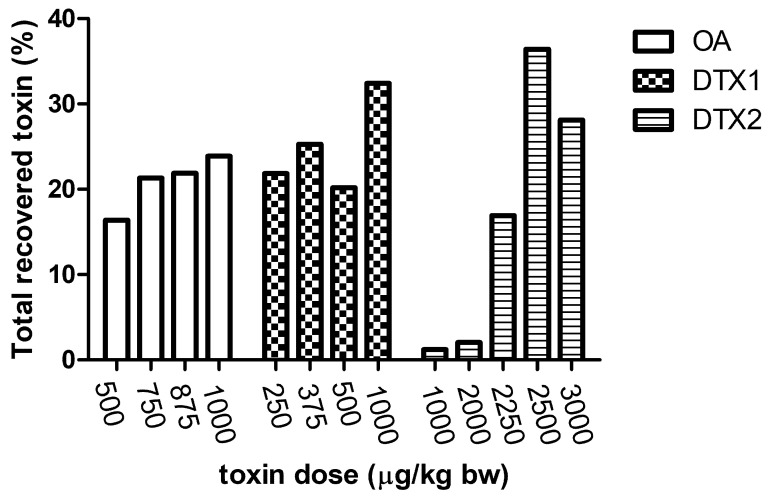
Toxin recovery: % OA, DTX1, or DTX2 detected 24 h after administration of the dose indicated.

As depicted in Figure 6, around 20% of the dose was recovered at 24 h after a single dose administration of OA. This percentage is similar for DTX1, but it should be highlighted that a recovery of 32% was found for the dose of 1000 µg/kg. In contrast, following a single dose of DTX2, the mean recovery of toxins accounted for 2% of the total dosages 1000 and 2000 µg/kg. The recoveries from the other DTX2 doses were higher and reached 35% (2500 µg/kg).

## 3. Discussion

The worldwide incidence of diarrheic shellfish toxins poses a threat to public health, with a consequent marine environment effect and great economic impact on the seafood industry [10,29,30]. In vivo toxicity differences were observed for the toxins of this group, both intraperitoneally [14,31] and orally [15]. In toxicity studies, it is important that the route of administration of the toxins is appropriate to the human situation; therefore, oral administration has been recently proposed as the most suitable for the study of these aquatic toxins and others naturally acquired by this route [14]. Besides, toxins’ tissue distribution could be a starting point to understand their behavior in the organism. For this purpose, different doses of OA, DTX1, and DTX2 were administered by oral route in mice and they were detected by LC/MS/MS analysis in gastrointestinal organs and different fluids.

Our results show that, after acute oral administration in mice, symptoms were observed after 1 h, with completely recovery within 24 h [19,20]. OA, DTX1, and DTX2 passed through the gastrointestinal barrier, are distributed across organs, accumulate in the stomach and the small and large intestines, and are eliminated in feces and in stomach and intestine contents. After each toxin administration, the recorded nonspecific systemic signs and symptoms included apathy, piloerection, squint-eyes, spams, cyanosis, on hind legs, and dyspnea, which was in accordance with previous findings after acute oral OA administration. [32]. One hundred percent of mortality was only reached with 3000 µg/kg DTX2, while all mice survived with the lowest concentrations of DTX1 (250 and 375 µg/kg) and DTX2 (1000 µg/kg). However, the representative symptom of DSP is diarrhea, which appeared soon after oral administration of toxins such as OA and DTX1 (30 min–2 h). This indicates the rapid effect of the toxins that was previously associated with fast absorption [15,19,26,27]. Related to that, pathological changes by OA were previously detected within 2 h [20]. In an in vivo situation, intestinal peristalsis prevents long exposure times within the same intestine section [33]. However, DSP toxins cause an alteration in intestinal motility that enables their intestinal absorption and enterohepatic circulation [34].

Therefore, DSP toxins pass through the gastrointestinal barrier to the bloodstream [18,20,32,34]. An analysis of the passage of these toxins through the gut barrier indicates that their absorption could be related to the ability to modify cellular structures such as the cytoskeleton or tight junctions. These changes were previously evaluated by electron and confocal microscopy, confirming modification in the intestinal microvilli as well as the redistribution of occludins, an important protein of tight junctions [15,27]. These alterations affect the barrier function of the intestinal epithelium and therefore could be involved in the changes in absorption of the different analogs. In our hands, these toxins were detected in blood 24 h after oral administration, with higher plasma levels of OA than those of DTX1. However, the rates of gastrointestinal transfer to the bloodstream orally appear to be low compared to blood levels attained by i.p. Similar differences between oral and i.p. administration were recently reported using other toxins such as mycrocystin [35].

Via blood circulation, the toxins are able to reach a variety of organs. Nevertheless, DSP toxin biodistribution was not uniform for all tissues. Analogs of OA, DTX1, and DTX2 vary in C31 and C35 methylation and stereochemistries [36,37]. The structure–activity relationship in the OA toxin group was previously studied by Twiner et al. [6]. In our experiments, small variations in OA and DTX1 toxin structures, specifically methylation at C35, only mildly affected body tissue accumulation whereas a lack of methylation at C31 along with methylation at C35 provided structural bases not only for the reduced toxicity but also for changes in tissue accumulation of DTX2. We focused on gastrointestinal organs in which OA-induced morphological changes were already described [38]. Within 24 h posttreatment, the three toxins were detected by LC-MS/MS analysis in all of the examined organs (liver, stomach, and small and large intestines). Even though the amount of compound depends on the administered dose, the highest concentration of OA and DTX1 were found in stomach with results similar to those reported by nearby doses of OA [32], while lower concentrations were found in liver. Therefore, these toxins could be retained in the stomach, which may explain the lesions noted in some studies at the stomach mucosa and submucosa in mice treated with OA [38,39]. In addition, OA and DTX1 seem to be accumulated in the intestine wall, which could be in connection with their gastrointestinal injuries [6,15,40]. The direct or local action of OA and DTX1 in the intestine may be the trigger for hypersecretion, inflammation, and diarrhea [20,23,41]. Our data revealed toxicokinetic differences between OA and DTX1 since, at the same dose of toxin (1000 µg/kg), the level of OA was higher (408 ng/g and 2340 ng/g) than DTX1 (182 ng/g and 1009 ng/g) in both the small and large intestines. Interestingly, the amount of DTX2 (142 ng/g) in the stomach was very low compared to OA (4540 ng/g) or DTX1 (3006 ng/g). This behavior was the same in the tissues of the liver and the small and large intestines and could indicate relatively low absorption of DTX2 from the gastrointestinal tract, as was previously suggested [20]. In agreement with that, studies of transepithelial permeability using an in vitro intestinal model demonstrated the very low ability of DTX2 to cross the intestinal barrier [42]. Besides, DTX2 was found in feces up to 24 h, with this excretion being important for the toxin. High fractions of the administered doses of OA and DTX1 were found in feces and were also recovered from the stomach content. Particularly, OA was detected in feces 2–3 h after administration, suggesting that this excretion was a fast and predominant route of toxin elimination from the organism. The recovery of the administered doses up to 1000 µg/kg OA (18–23%) and DTX1 (20–32%) was higher than that of DTX2 (1–2%). Only with doses of DTX2 higher than 2250 µg/kg was the total recovery around 30%, indicating that the absorption rate of DTX2 is low [27].

The present experiments confirmed some data about organ distribution and excretion of OA previously published [32,34], indicating that, 24 h after oral administration, OA was detected in urine, gastrointestinal contents, and gastrointestinal tissues. However, so far, relatively little information has been collected about the organ distribution of DTX1 and DTX2. Our data suggest a different toxicokinetic pathway between OA, DTX1, and DTX2 and incomplete absorption of the toxins. This could be due to the rapid induction of diarrhea and the consequent elimination of a considerable quantity of OA and analogs by feces or intestinal content [12,14,15,27]. DSP toxins could also change to other metabolites. Recent papers focused on the rapid esterification of DSP toxins with fatty acids in mollusks [43,44]. The presence of diol esters of DSP toxins in dinoflagellates was also recently evaluated; moreover, a different intraperitoneal toxicity of esters compared with free toxins was suggested [45]. The metabolism of OA by NADPH-dependent enzymes present in human or rat liver S9 fractions was already reported and resulted in different toxic effects [46]. Nevertheless, in previous studies, excreted OA and DTX1 were on the free form, not esters, and toxins were particularly in connection with injuries [15,20,34,38]. Therefore, this study did not include toxin ester analysis. A more detailed investigation will be required to confirm any toxin biotransformation in mice.

It should be noted that the amount of DTX2 in the small intestine was higher than in large intestine, opposite to OA and DTX1. This information is interesting but partial since the toxins could be excreted or accumulated in other organs.

Then, the in vivo differences in the toxicity of DTX2 versus OA and DTX1 lie in the less pharmacological potency [6,7,27,47] but could also be associated with scarce gastrointestinal absorption and low accumulation in intestinal tissues.

## 4. Materials and Methods

The toxins OA and DTX1 were provided by the National Research Institute of Fisheries Science (NRIFS) from the Fisheries Research and Education Agency (Yokohama, Japan). OA and DTX1 isolated from toxic dinoflagellate *Prorocentrum lima* [48] were quantified by the PULCON method [49] on a quantitative NMR with external standards. The purities (purity > 95%) of both toxins were also confirmed by NMR spectroscopy. DTX2 (purity > 98%) was a certified reference material (CRM) supplied by Laboratorio CIFGA S.A. (Lugo, Spain). Stock solutions of the toxins were diluted to target doses in 0.9% saline solution and administered orally at 10 mL/kg bw in mice.

All chemicals employed were HPLC or analytical grade from Sigma-Aldrich Quimica S.A. (Madrid, Spain).

### 4.1. In Vivo Assays and Animal Conditions

In vivo assays were performed according to the Organisation for Economic Co-operration and Development (OECD) standardized method 4-level up and down procedure, which includes the reduction of the number of animals in the three Rs principle (replace, reduce, and refine) [50]. In all cases, the starting dose was 1000 µg/kg bw. The dose of the next level in the design depended on the toxicity in the previous level, and the number of mice was increased at each dosage level, as was previously described [27].

Briefly, four-week-old female C57BL/6J mice weighing 20 g were fasted overnight and, at 9 a.m., were weighed again. Then, they received a dose of one of the toxins (OA, DTX1, or DTX2) by oral gavage at the moment in which food and drink were provided ad libitum. The experiment concluded 24 h after toxin administration with euthanasia of the surviving animals.

For the urine and fecal excretion studies, urine and feces were collected at time points of 1, 3, 6, 9, 12, and 24 h after toxin administration. All samples were stored frozen at −20 °C until analysis.

The whole blood samples were collected in heparinized tubes at the end of the experiment and centrifuged at 3000× *g*. Plasma was separated and stored frozen at −20 °C until analysis.

All animal procedures described in the manuscript were carried out in conformity to European legislation (EU directive 2010/63/EU) and Spanish legislation (Real Decreto 53/2013, Decreto 296/2008) and to the principles approved by the Institutional Animal Care Committee of the Universidad de Santiago de Compostela under the procedure code: 01/17/LU-002 (approved on 22 September 2017).

### 4.2. LC/MS/MS Analysis of Mice Organs

All animals in the study were subjected to a full necropsy. Organs from the gastrointestinal tract were collected after mice death or euthanasia to evaluate toxin distribution. Organs were stored at −80 °C until LC/MS/MS analysis. Then, the organs were weighed and extracted with methanol, as was previously described [27]. Briefly, 0.1 g of homogenized sample was extracted by adding 400 µL of methanol, and after 60 s of vortex mixing and 30 s of sonication, the mixture was centrifuged at 10,000× *g* for 10 min at room temperature. The supernatant was transferred to a microtube, and the remaining pellet was extracted two more times. Then, the combined supernatants were evaporated and reconstituted in 100 µL of methanol to finally be mixed with 40 µL of methanol (vortex-mixed for 30 s) and 10 µL of trichloroacetic acid 10% for protein precipitation (vortex-mixed for 30 s). Then, 50 µL of CH_3_CN was added (vortex-mixed for 1 min), and after centrifugation at 14,500× *g* for 10 min at room temperature, the mixture was filtered through 0.22 µm into HPLC vials for analysis by LC/MS/MS, with two replicates of the same sample.

The urine sample extraction protocol was performed according to Guada et al. (2013) [51] and modified by Abal et al. (2017) [27]. Briefly, 40 mL of methanol was added to 100 mL of urine homogenized samples and vortex-mixed for 30 s. Then, for protein precipitation, the samples were mixed with 10 mL of 10% trichloroacetic acid for 30 s. Finally, 50 mL of CH_3_CN was added and vortex-mixed for 1 min. Samples were then centrifuged at 14,500× *g* for 10 min at room temperature, and the extract was filtered (0.22-mm centrifugal filter, Merk Millipore, Billerica, MA, USA). Five microliters of this sample were injected into the LC/MS system, with two replicates of the same sample. Stomach, small intestine, and large intestine content extractions were performed following the urine sample extraction protocol.

Fecal sample extraction was performed according to Abal et al. (2017) [27]. Feces were weighed and extracted by adding 400 mL of methanol to 0.1 g of the homogenized sample. After 60 s of vortex mixing and 30 s of sonication, samples were centrifuged at 10,000× *g* for 10 min at room temperature, and the supernatant was transferred to an eppendorf. After three extraction procedures, the total supernatant was evaporated and reconstituted in 100 mL of methanol. The subsequent steps of the extraction protocol were common to urine sample extraction.

The blood extraction protocol was performed according to Abal et al. (2018) [15]. Briefly, 800 μL of 75% methanol was added to 200 μL of the intracardiac blood sample and vortexed for 1 min. The mixture was transferred to an ultrafiltration spin column and centrifuged at 3000 rpm for 30 min. Then, the ultrafiltered solution was evaporated and reconstituted with 200 μL of methanol 100%. Finally, samples were filtered by 0.22 μm for 10 min at 14,500× *g* at room temperature, and 5 μL was subjected to LC-MS/MS, with two replicates of the same sample.

#### LC/MS/MS Conditions

Analysis of the organ extracts was performed on a 1290 Infinity ultra-high performance liquid chromatography system coupled to a 6460 Triple Quadrupole mass spectrometer (Agilent Technologies, Waldbronn, Germany), as previously described [15,27]. The mass spectrometer was operated in Multiple Reaction Monitoring (MRM) in negative mode, analyzing all OA, DTX1, and DTX2 transitions known, using the highest intensity transition for quantification (*m*/*z* 803.5 > 255.2 OA, *m*/*z* 817.5 > 255.2 DTX1, and *m*/*z* 803.5 > 255.1 DTX2) and one transition for confirmatory purposes (*m*/*z* 803.5 > 113.2 OA; *m*/*z* 817.5 > 113.0 DTX1, and *m*/*z* 803.5 > 151.0 DTX2).

All parameters were optimized with accurate well-characterized OA, DTX1, and DTX2 standards in order to achieve the maximum level of sensitivity. Cell accelerator voltage (CAV) was 4 V, and the fragmentor was 320 V. Furthermore, collision energy (CE) value was optimized for each transition: *m*/*z* 803.5 > 255.2 (CE = 50 V) and 803.5 > 113.2 (CE = 66 V) for OA, *m*/*z* 817.5 > 255.2 (CE = 54 V) and 817.5 > 113.0 (CE = 70 V) for DTX1, and *m*/*z* 803.5 > 255.1 (CE = 56 V) and 803.5 > 151.0 (CE = 56 V) for DTX2.

Toxin standards were used for toxin calibration in the range 0.19–100 ng/mL. The estimated limit of detection (LOD) based on a signal-to-noise ratio of 3 (S/N = 3) and the limit of quantification (LOQ) considering a signal-to-noise ratio of 10 (S/N = 10) were 0.2 ng/mL and 1.3 ng/mL, respectively, for both OA and DTX1, and 0.7 ng/mL (LOD) and 2.33 ng/mL (LOQ) for DTX2.

### 4.3. Statistical Analysis

The results were analyzed by 1-way ANOVA complemented with Newman–Keuls Multiple Comparison Test. *p* ≤ 0.05 was considered statistically significant.

## 5. Conclusions

The results help us understand the different distribution pattern of DSTs in gastrointestinal organs. Absorption of DSP toxins from the gastrointestinal tract and accumulation in the organs were different and dose-dependent, suggesting a distinct toxicokinetic pathway between OA, DTX1, and DTX2. It can be speculated that the low absorption of DTX2 may reduce its in vivo effects. From a toxicological point of view, it is important to highlight that OA and DTX1 are quickly absorbed orally and can accumulate in the stomach and the small and large intestines, which is associated with its rapid and acute effects, even though the toxic potency cannot be excluded. This preliminary study provides useful information to better assess human health risks associated with DSP toxin-contaminated seafood.

## Figures and Tables

**Table 1:** Lethality (%) of mice after Okadaic acid (OA), dinophysistoxin-1 (DTX1), or dinophysistoxin-2 (DTX2) administration by oral gavage.

Toxin	Dose (µg/kg bw)	Lethality (%)	Number Mice
OA	1000	67	3
	875	67	9
	750	43	7
	500	40	5
DTX1	1000	67	3
	500	60	5
	375	0	9
	250	0	7
DTX2	3000	100	5
	2500	86	7
	2250	44	9
	2000	33	3
	1000	0	3

**Table 2:** Symptoms registered in mice after OA, DTX1, or DTX2 administration by oral gavage.

	Appearance of Symptoms (%)												
	OA (µg/kg bw)				DTX1 (µg/kg bw)				DTX2 (µg/kg bw)				
Symptom	1000	875	750	500	1000	500	375	250	3000	2500	2250	2000	1000
diarrhea	66.67	100	57.14	100	66.67	80	66.67	71.43	100	100	33.33	66.67	66.67
apathy	100	88.89	100	100	100	100	88.89	85.71	100	100	44.44	66.67	66.67
piloerection	100	55.56	42.86	20	33.33	40	44.44	28.57	40	14.29	11.11	33.33	66.67
squint-eyes	100	77.78	57.14	60	100	80	77.78	57.14	20	0	0	0	33.33
spasms	33.33	22.22	28.57	0	33.33	20	22.22	28.57	0	0	11.11	33.33	33.33
cyanosis	66.67	88.89	57.14	0	66.67	80	66.67	0	60	85.71	33.33	66.67	0
on hind legs	0	44.44	14.29	60	0	20	22.22	14.29	0	14.29	0	0	33.33
dyspnea	0	0	14.29	0	33.33	0	0	0	40	14.29	0	0	0

**Table 3:** Concentration of toxin (ng/g tissue) in gastrointestinal organs from mice treated with 1000 µg/kg body weight (bw) doses (mean ± SEM): 1-way ANOVA–Newman–Keuls Multiple Comparison Test was used, and * *p* ≤ 0.05 was considered statistically significant.

	Liver	Stomach	Small Intestine	Large Intestine
OA	96 ± 23	4540 ± 1326	408 ± 271	2340 ± 706
DTX1	93 ± 6	3006 ± 782	182 ± 27	1009 ± 83
DTX2	21 ± 11 *	142 ± 105 *	75 ± 38	176 ± 120 *

**Table 4:** Toxin content (ng) quantified in fluids collected from the stomachs and the small and large intestines of mice treated with 1000 µg/kg bw doses (mean ± SEM): 1-way ANOVA–Newman–Keuls Multiple Comparison Test showed no significant differences between OA and DTX1. *p* ≤ 0.05 was considered statistically significant.

	Stomach Content	Small Intestine Content	Large Intestine Content
OA	2112 ± 365	151 ± 135	397 ± 193
DTX1	4468 ± 1802	321 ± 50	341 ± 72
DTX2	-	30 ± 1.3	0.19 ± 0.092

## Data Availability

The data presented in this study are available on request from the corresponding author.
